# Grindelia Robusta in Asthma

**Published:** 1881-06

**Authors:** 


GRINDELIA ROBUSTA IN ASTHMA.—Dr. T. M. Rochester has recently read a paper before the Kings County Medical Society, in which he states that he has used this article in over sixty cases of asthma and found relief from it in all of them. In some the relief was not of long duration. But in most cases it seemed to be permanent. He gave half-drachm doses of the fluid extract every fifteen minutes until relief was obtained, and in the intervals fifteen to twenty-drop doses every four to six hours. Its use should be continued for from a week to ten days. It has also been used quite successfully in the same disease in the U. S. Marine Hospital, at Portland, Me.

